# Support Vector Machine for Regional Ionospheric Delay Modeling

**DOI:** 10.3390/s19132947

**Published:** 2019-07-04

**Authors:** Zhengxie Zhang, Shuguo Pan, Chengfa Gao, Tao Zhao, Wang Gao

**Affiliations:** 1 School of Transportation, Southeast University, Nanjing 210096, China; 2 School of Instrument Science and Engineering, Southeast University, Nanjing 210096, China; 3 Key Laboratory of Micro-Inertial Instrument and Advanced Navigation Technology, Ministry of Education, Nanjing 210096, China

**Keywords:** SVM, ionosphere model, TEC, single-frequency PPP, BPNN

## Abstract

The distribution of total electron content (TEC) in the ionosphere is irregular and complex, and it is hard to model accurately. The polynomial (POLY) model is used extensively for regional ionosphere modeling in two-dimensional space. However, in the active period of the ionosphere, the POLY model is difficult to reflect the distribution and variation of TEC. Aiming at the limitation of the regional POLY model, this paper proposes a new ionosphere modeling method with combining the support vector machine (SVM) regression model and the POLY model. Firstly, the POLY model is established using observations of regional continuously operating reference stations (CORS). Then the SVM regression model is trained to compensate the model error of POLY, and the TEC SVM-P model is obtained by the combination of the POLY and the SVM. The fitting accuracies of the models are verified with the root mean square errors (RMSEs) and static single-frequency precise point positioning (PPP) experiments. The results show that the RMSE of the SVM-P is 0.980 TECU (TEC unit), which produces an improvement of 17.3% compared with the POLY model (1.185 TECU). Using SVM-P models, the positioning accuracies of single-frequency PPP are improved over 40% compared with those using POLY models. The SVM-P is also compared with the back-propagation neural network combined with POLY (BPNN-P), and its performance is also better than BPNN-P (1.070 TECU).

## 1. Introduction

The ionosphere is a part of Earth’s upper atmosphere, which is ionized by solar radiation and contains a large number of free electrons. These free electrons are sufficient to affect the radio propagation and cause ionospheric delays of global navigation satellite system (GNSS) signals, which are the main sources of errors in GNSS applications. They seriously affect the performance of navigation and positioning, and must be effectively reduced or eliminated [1]. With the development of GNSS technology and construction of ground-based GNSS stations, GNSS brings great opportunities for the development of the ionosphere research. GNSS has become one of the most important methods of ionospheric monitoring, with high accuracy, wide range, and continuous operation [2].

Total electron content (TEC) is an important parameter to describe the characteristics of the ionosphere. The establishment of the ionosphere model based on GNSS data can effectively characterize the distribution and variation of TEC in the region [3,4]. Therefore, the accuracy of the ionosphere model directly affects the receiver positioning performance [5,6,7]. The two-dimensional polynomial (POLY) model is most commonly used in ionospheric fitting, which can obtain a good accuracy with enough date in the stable period of the ionosphere. However, due to the complex ionospheric changes, it is difficult to obtain high-accuracy in a wide range of the region. Therefore, how to improve the capability of the ionosphere model is the focus of current research.

In the past two decades, artificial neural networks(ANN) have been widely used in ionosphere research and have shown good results in ionospheric modeling, tomography, and prediction [8,9,10]. Many scholars have conducted researches on the application of ionosphere with ANN and made many research achievements. Habarulema [11] used the back propagation neural network (BPNN) method to construct a regional TEC model using a single station observation in turn, which is suitable for small areas. McKinnell [12] established a high-latitude ionospheric prediction model by selecting the optimal NN training parameters. Fan [13] proposed a vertical total electron content (VTEC) model based on generalized regression neural network (GRNN) with high extrapolation accuracy. Hu [14] proposed a fusion model of the VTEC combined two-dimensional model and BPNN. Ghaffari [15] applied the wavelet neural network(WNN) and particle swarm optimization (PSO) algorithm to the ionospheric tomography in the Iranian region. Although many scholars have done a lot of research on neural networks, there are still many problems in the ANN, such as the choice of network parameters, the setting of the network structure, and the uncertainty of the output results.

Support Vector Machine (SVM) is one machine learning algorithm based on the principle of structural risk minimization and VC dimension (Vapnik Chervonenkis dimension) theory for classification, regression, and other learning tasks. SVM was first proposed by Vapnik [16] in 1995 and has been used in text categorization, pattern recognition, cluster analysis, etc. SVM can use training samples effectively and has great generalization ability [17,18,19]. SVM provides a method to construct a mapping into a higher-dimensional feature space by using a kernel function, such as linear, polynomial, sigmoid, and radial basis function(RBF). At present, in the ionosphere field, the SVM algorithm is mostly used to solve the ionosphere prediction problem [20,21]. Ban [22] used the SVM algorithm to establish the low-latitude storm ionosphere foF2 model, which can detect the ionospheric disturbance better and has improved compared with the empirical model. Akhoondzadeh and Liu used SVM to analyze the TEC time series before and after the earthquake, and proved the effectiveness of SVM in seismic anomaly detection [23,24]. However, there is little research on using SVM for ionosphere modeling correction.

This paper proposes an SVM-based ionosphere model (SVM-P) aiming at the problem of high-accuracy modeling of the ionosphere, considering the limited accuracy of the 2D TEC model and the good nonlinear regression performance of the SVM. Based on the POLY model, the SVM regression is used to establish the TEC error prediction for model correction and optimizing the model accuracy. The accuracy of the SVM-P model is analyzed by GNSS data from continuously operating reference stations (CORS), compared to the POLY model and BPNN-POLY (BPNN-P) model. The effectiveness of the method is verified by the single-frequency PPP method.

The rest of this paper is organized as follows: Section 2 describes the principle of ionosphere POLY model and SVM regression. In Section 4, the process of modeling with SVM and POLY are introduced. In Section 3, the modeling performance for POLY, SVM-P, and BPNN-P are analyzed by model accuracy and single-frequency PPP. The discussion and conclusion will be given in Section 4 and Section 5, respectively.

## 2. Materials and Methods

### 2.1. Ionosphere Polynomial Model

According to the dispersion characteristics of the ionosphere, the ionospheric errors caused by GNSS signal delay are related to frequencies. Using the dual-frequency receiver, the ionospheric TEC can be extracted via a linear combination of observations [15], and the phase smoothing pseudo-range method is used to reduce the observation noise, which can be expressed as:

(1) $$VTEC=\frac{f_{1}^{2}f_{2}^{2}}{40.28mf(f_{1}^{2}-f_{2}^{2})}({\bar{P}}_{4}+b_{r}+b^{s})$$

where, VTEC represents vertical total electron content, $f_{1}$ and $f_{2}$ represent carrier frequencies, mf represents mapping function, ${\bar{P}}_{4}$ represents pseudo-range observation difference by smoothing, where $P_{4}=P_{2}-P_{1}$, $P_{1}$ and $P_{2}$ represent dual-frequency pseudo-range observations, $b_{r}$ and $b^{s}$ represent code biases of receiver and satellite respectively. Code bias can be considered as a constant in 1–2 days [25,26], which are corrected by the previous day’s estimation in this paper. 

It is generally assumed that all free electrons are all distributed in a thin shell of height H when modeling ionosphere using a 2D surface. In this paper, H is valued by 350 km. The location of the GNSS signal across the ionospheric shell is called the ionospheric pierce point (IPP) and is represented by geographic latitude and longitude [27]. Based on the VTEC observation of the CORS, the latitude, and longitude of the IPPs, a regional ionospheric VTEC PLOY model is represented as following [28]:

(2) $$VTEC=\sum_{i=0}^{m}\sum_{k=0}^{n}a_{i,k}{\left(\varphi -\varphi_{0}\right)}^{i}{\left(S-S_{0}\right)}^{k}$$

where, m and n are the maximum degrees, $a_{i,k}\left(i=0,1,\ldots ,m;k=0,1,\ldots ,n\right)$ are the (unknown) coefficients, S is sun angle at time t, $S-S_{0}=\left(\lambda -\lambda_{0}\right)+\left(t-t_{0}\right)$, φ and λ represent latitude and longitude of IPPs, $\varphi_{0}$ and $\lambda_{0}$ are the coordinates of the origin of the polynomial, which are the region center generally.

### 2.2. Support Vector Machine

**Figure 1:**
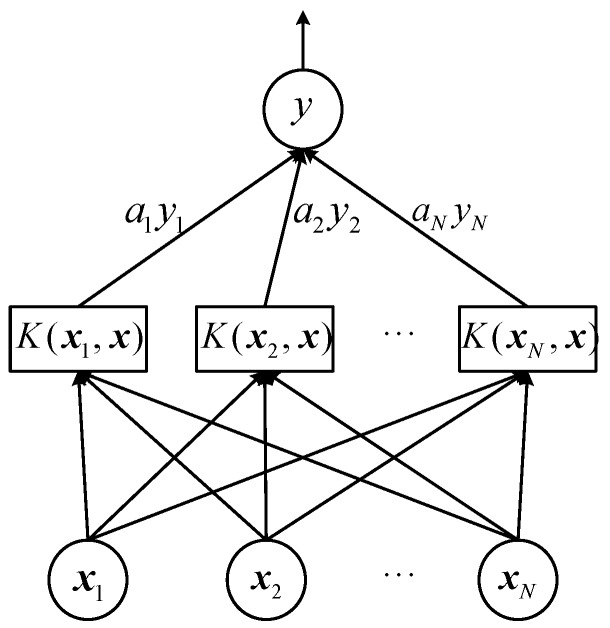
Support Vector Machine (SVM) algorithm structure: the feature vectors are input parameters and the target is the output parameter.

In this section, we will briefly introduce the principles and methods of SVM applied to regression problems. SVM shows good performance in classification and is considered easier to use than neural networks. Extending classification to regression issues, SVM has a similar structure with neural networks [29]. The process of the SVM training is shown in Figure 1.

Given a training set $D=\left\{\left(x_{1},y_{1}\right),\left(x_{2},y_{2}\right),\ldots ,\left(x_{N},y_{N}\right)\right\},x_{i}\in R^{d},y_{i}\in R$, where x is the *d*-dimensional feature vector, y is 1-dimensional target value. Due to the samples are not linearly separable, they are mapped to the higher-dimensional feature space by the nonlinear mapping function φ(x). The regression function can be converted to a convex optimization problem [30].

(3) f(x)=ω⋅φ(x)+b

(4) $$\min\frac{1}{2}{\left\|\omega \right\|}^{2}+C\sum_{i=1}^{N}\left(\xi_{i}^{\vee }+\xi_{i}^{\wedge }\right)$$

subject to:

(5) $$\{\begin{array}{c} -\varepsilon -\xi_{i}^{\vee }\leq y_{i}-\omega \cdot \varphi \left(x_{i}\right)-b\leq \varepsilon +\xi_{i}^{\wedge }\\ \begin{array}{c} \xi_{i}^{\vee }\geq 0\\ \xi_{i}^{\wedge }\geq 0 \end{array} \end{array}$$

where ω is the normal vector to the hyperplane, ‖·‖ represents L2 matrix norm, $\xi_{i}^{\vee }$ and $\xi_{i}^{\wedge }$ are the lower and upper boundary of slack variables, C is the penalty factor, and ε is the allowable error level of ε-Insensitive the loss function. The dimensions of φ(x) and ω are determined by kernel function. Adding the Lagrangian multiplier, the optimization problem described by Equation (4) is transformed into a dual problem. By solving the dual problem, the regression solutions are represented as follows:

(6) $$f\left(x\right)=\sum_{i=1}^{n_{SV}}\left(a_{i}^{\wedge }-a_{i}^{\vee }\right)K\left(x_{i},x\right)+b$$

(7) $$K\left(x_{i},x\right)=<\varphi \left(x_{i}\right),\varphi \left(x\right)>=\exp\left(-\gamma {\left\|x-x_{i}\right\|}^{2}\right)$$

where $n_{\mathrm{SV}}$ denotes the number of support vectors, $a_{i}^{\vee }$ and $a_{i}^{\wedge }$ Lagrangian multiplier, $K\left(x_{i},x\right)$ indicates kernel function, γ is kernel parameter. In this paper, the RBF kernel is adopted [31] as Equation (7), which replaces the inner product operation in higher-dimensional space, simplifies the approximation process of nonlinear regression and reduces the amount of computation.

### 2.3. Support Vector Machine (SVM) for Ionosphere Model Correction

**Figure 2:**
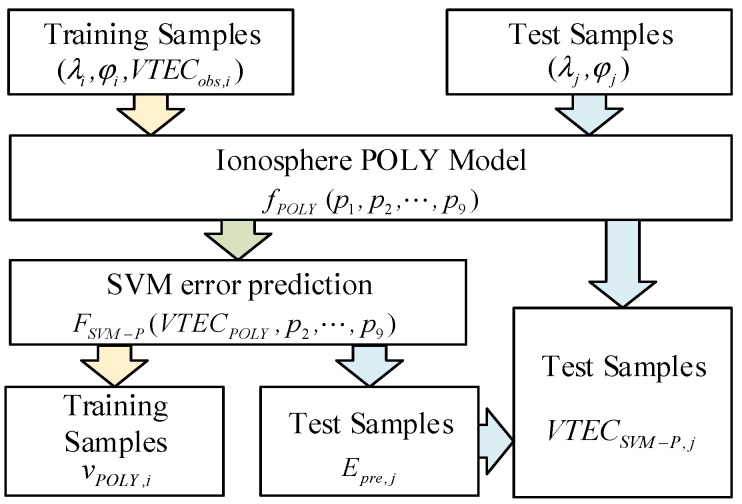
The flow chart of the Support Vector Machine polynomial (SVM-P) modeling process.

Based on the ionosphere POLY model, this paper proposes an SVM-based ionosphere model correction method named SVM-P. The SVM regression is used to establish an error prediction model to correct POLY. The specific implementation steps are shown in Figure 2.

#### 2.3.1. Establish the Ionosphere Polynomial Model

Using the dual-frequency ionospheric TEC observations of the CORS, the regional ionosphere POLY model is established according to Equation (2). In this paper, the maximum order of the POLY is selected: m=n=2, and 9 model coefficients $\left(a_{0,0},a_{0,1},\ldots ,a_{2,2}\right)$ need to be solved. For convenience, we use $p_{1},p_{2},\ldots ,p_{9}$ to represent the position parameters $(1,\Delta \varphi ,\Delta S,\;\Delta \varphi^{2},\Delta S^{2},\Delta \varphi \Delta S,\Delta \varphi^{2}\Delta S,\Delta \varphi \Delta S^{2},$ $\Delta \varphi^{2}\Delta S^{2})$ of IPPs, where $\Delta \varphi =\;\varphi -\varphi_{0}$, $\Delta S=S-S_{0}$. The POLY model and its residual of each IPPs can be calculated as follows:

(8) $$VTEC_{POLY,i}=f_{POLY}(p_{1,i},p_{2,i},\cdots ,p_{9,i})=a_{0,0}p_{1}+a_{0,1}p_{2,i}+a_{1,0}p_{3,i}+\cdots +a_{2,2}p_{9,i}$$

(9) $$v_{POLY,i}=VTEC_{obs,i}-VTEC_{POLY,i}$$

where i=1,2,…,N represents the number of training samples, $VTEC_{POLY}$ is the VTEC of IPP calculated by POLY, $VTEC_{obs}$ is the VTEC observation, which is used as a true value in this paper, $v_{POLY}$ is the residual of POLY. According to the least square criterion, the coefficients can be solved to establish the POLY model.

#### 2.3.2. Support Vector Machine (SVM) Regression for Residuals

The training samples are constructed to obtain the SVM regression model. The input parameters include: 1 VTEC value of POLY($VTEC_{POLY}$) and 8 IPPs’ position information $p_{2},p_{3},\ldots ,p_{9}$. The output target is the residual of the POLY model ($v_{POLY}$). Since the SVM regression model is nonlinear and cannot be expressed by a certain function, it is assumed that the regression function is represented as follow.

(10) $$E_{pre}=F_{SVM}\left(VTEC_{POLY},p_{2},p_{3},p_{4},p_{5},p_{6},p_{7},p_{8},p_{9}\right)$$

where, $F_{SVM}$ represents SVM regression function, $E_{pre}$ represents the prediction of POLY model residual from SVM regression, which is the correction for POLY. When the training of SVM is over, we will get an SVM regression function in the whole region.

#### 2.3.3. Support Vector Machine Polynomial (SVM-P) for Ionospheric Delay Correction

According to the trained SVM regression, the $E_{pre}$ can be calculated to correct POLY. Combined with the $VTEC_{POLY}$ form POLY model, the prediction value of VTEC $VTEC_{SVM-P}$ is expressed as:

(11) $$VTEC_{SVM-P}=VTEC_{POLY}+E_{pre}$$

By Equations (8), (9), and (11), the ionospheric delay correction $VTEC_{SVM-P}$ can be obtained in any position of IPP via the SVM-P model.

## 3. Experiments and Results

### 3.1. Data Processing

**Figure 3:**
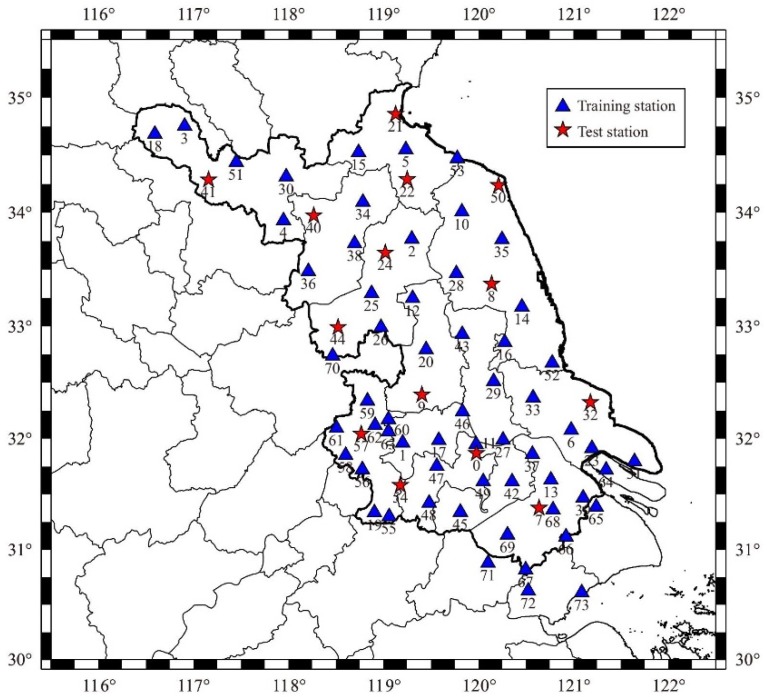
The distribution of JSCORS.

The training and test datasets are collected from CORS of Jiangsu Province (JSCORS), China, which include 1-day observations of 74 stations in DOY 324, 2010 (interval 30 s). The distribution of stations shown in Figure 3. 60 stations represented by the blue triangle (called training stations) are used for ionosphere modeling and SVM training. Distributing evenly throughout the region, 14 stations represented by the red star (called test stations), which do not participate in modeling and training, are used to test the accuracies of the models. Two stations numbered 32 and 50 (BTRD and GTBH), a part of test stations, are used for single-frequency PPP positioning experiments.

**Figure 4:**
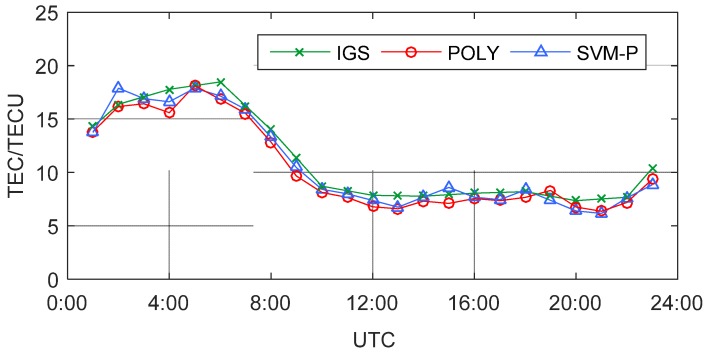
VTEC time series of IGS, polynomial (POLY), and Support Vector Machine Polynomial (SVM-P) in the regional center zenith direction.

Firstly, the training data is used for regional POLY modeling and to solve the POLY’s coefficients, and the 23 hourly models are obtained (from 1:00 to 23:00, 0:00 and 24:00 are excluded). Then calculate the model residual $v_{POLY}$. Combine the residual $v_{POLY}$, the POLY model value $VTEC_{POLY}$, and the position information of the IPP to constitute training samples. Part of the training samples are shown in Table 1. Next, the SVM regression is used for the error prediction. Finally, calculate the error prediction value $E_{pre}$ and the SVM-P model value $VTEC_{SVM-P}$. The VTEC time series in the regional center zenith direction are calculated by the POLY model and the SVM-P model, compared with the International GNSS Service (IGS) ionospheric product, as shown in Figure 4.

In Figure 4, the trends of the three series are coincident, indicating that there is no significant deviation among the POLY model, the SVM-P model, and the IGS product. Affected by solar radiation, the maximum of TEC in a day is about 19 TECU from 4:00 to 6:00 UTC (12:00 to 14:00 LT), and ionosphere is relative calm during the night, basically around 8 TECU. Besides, the POLY model and the SVM-P model use regional observations, which can represent small-scale changes in TEC more prominently.

### 3.2. Parameters Selection of Support Vector Machine (SVM)

Since the samples are mapped to a higher-dimensional space during the SVM training process, the parameters of the kernel function directly affect the performance of the SVM regression. The parameter γ determines the complexity of the sample mapping, and C achieves the balance among the model accuracy, the algorithm calculation, and the generalization ability. In this paper, the grid search method is used to compare and analyze the influence of RBF kernel parameters γ and C on regression. The correlation coefficient (CC) and the root mean square error (RMSE) are used as the criteria of parameter selection to search for optimal parameters. CC and RMSE can be represented as:

(12) $$CC=\frac{n\sum E_{pre}v_{POLY}-\sum E_{pre}\sum v_{POLY}}{\sqrt{n\sum E_{pre}^{2}-{\left(\sum E_{pre}\right)}^{2}}-\sqrt{n\sum v_{POLY}^{2}-{\left(\sum v_{POLY}\right)}^{2}}}$$

(13) $$RMSE=\sqrt{\frac{\sum {\left(E_{pre}-v_{POLY}\right)}^{2}}{n}}$$

where n is the number of test samples. A data set is selected to analyze the relationship between the combination of kernel parameters and SVM regression performance.

**Figure 5:**
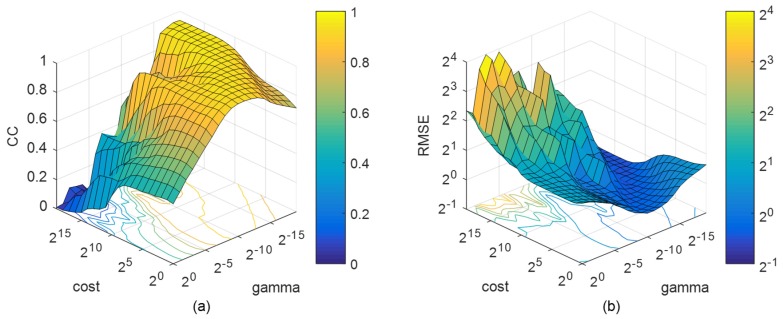
Grid search for kernel parameters in Support Vector Machine (SVM) regression. (**a**) correlation coefficient (CC); (**b**) root mean square error (RMSE).

Figure 5a shows the relationship between CC and the kernel parameters γ and C, and the impact of kernel parameters on the SVM performance is very significant. When one of the parameters is fixed, the CC will increase first and then decrease with the change of the other parameter. Finally, the optimal parameters converge in a peak space in yellow in the grid. The RMSE in Figure 5b shows an opposite trend with CC, as a valley space, but the range of optimal parameters is roughly the same. The combination of parameters in the optimal space can satisfy the accuracy requirements of CC and RMSE at the same time. In addition, the gradient of CC and RMSE is small in the optimal space, which indicates that SVM is not sensitive to the combination. Therefore, the optimal combination of parameters (C,γ) is (2^6^, 2^−14^).

### 3.3. Comparisons of Model Accuracies

We also use the BPNN algorithm combined with POLY (BPNN-P) to compare modeling results with POLY and SVM-P, which have the same process of modeling as SVM-P. The BPNN-P has 9 input nodes, a single hidden layer with 20 nodes, and 1 output node. The activation function and the learning rate are selected by sigmoid and 0.01. 

**Figure 6:**
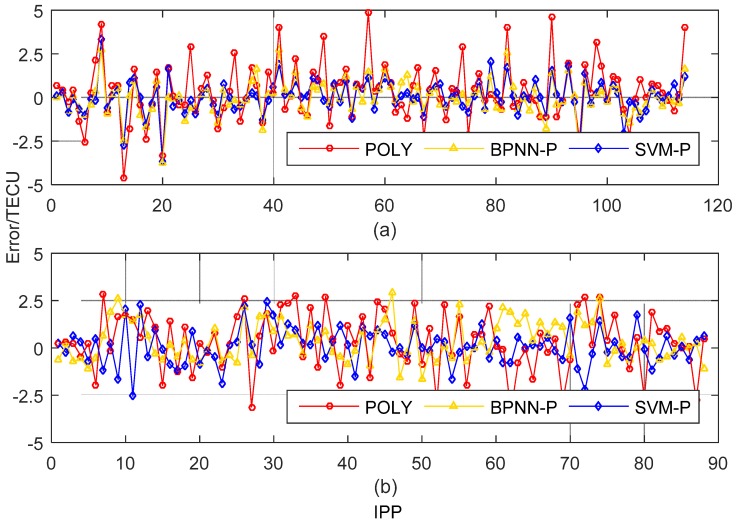
Total electron content (TEC) Model residuals of the test samples at two moments. (**a**) 3:00 UTC; (**b**) 19:00 UTC.

**Figure 7:**
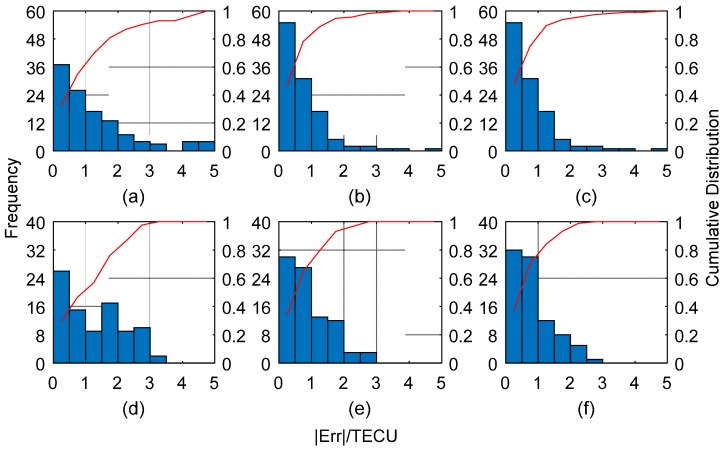
Residuals distribution statistics. (**a**–**c**) Residuals of polynomial (POLY), back propagation neural network combined with POLY (PBNN-P), and Support Vector Machine Polynomial (SVM-P) at 3:00 UTC with 115 samples. (**d**–**f**) Residuals of POLY, PBNN-P, and SVM-P at 19:00 UTC with 88 samples.

The data of test stations are analyzed for the model accuracies. Figure 6 shows the model residuals of the test samples at 3:00 and 19:00. Comparing the three series of residuals, the residuals of SVM-P and BPNN-P fluctuate stably around 0, which indicates SVM-P and BPNN-P have higher accuracies than POLY. From Figure 7, the distribution of |Err| (the absolute value of model residual) of SVM-P is more concentrated than BPNN-P and POLY. With |Err|<1.5TECU, the cumulative distribution of SVM-P is 89.6% at 3:00, greater than 88.7% in BPNN-P, and 69.6% in POLY. At 19:00, 84.1% in SVM-P is greater than 79.5% in BPNN-P and 56.8% in POLY. This illustrates that SVM-P has a good performance in error prediction, and improve the accuracy of the ionosphere model.

**Figure 8:**
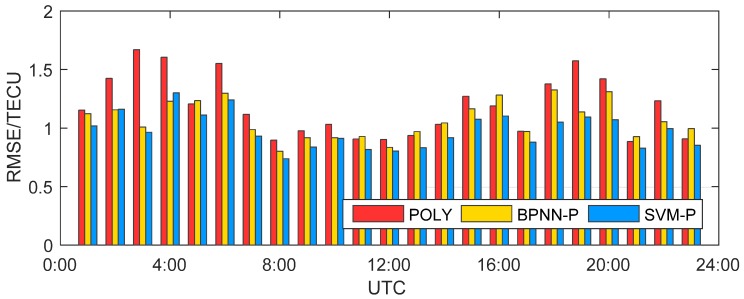
Model root mean square errors (RMSEs) of polynomial (POLY), back propagation neural network polynomial (BPNN-P), and Support Vector Machine polynomial (SVM-P) at 23 hourly moments.

The RMSEs of the models are shown in Figure 8, and the statistical results are shown in Table 2. Because the ionospheric activity is different at each moment and the model is greatly affected by the distribution of visible satellites and IPPs, the accuracies of the models are not uniform within one day, but all RMSEs are better than 1.7 TECU. Compared with POLY, the SVM-P model can improve the accuracy of the model. With the low accuracy of the POLY (RMSE is more than 1 TECU), the improvement effect is obvious, and the maximum can up to 42.3%. When the POLY has reached a high accuracy, the SVM-P can also increase slightly at least 6.1%. BPNN-P has roughly the same effect with SVM-P, but the accuracy is not as high as it. Even at some moments, BPNN-P is worse than POLY, such as 5:00 and 11:00. The mean RMSEs of POLY, BPNN-P, and SVM-P in one day are 1.185 TECU, 1.070 TECU, and 0.980 TECU, respectively from Table 2. It means the SVM-P can effectively reduce ionospheric modeling errors, and the improvement is 17.3% compared to POLY.

### 3.4. Single-Frequency PPP

In order to verify and compare how ionosphere models take effects on single-frequency PPP, station BTRD and GTBH are performed static single-frequency PPP positioning. The estimated parameters X in PPP include:

(14) $$X=[x,y,z,dt,Trop,N_{1},\ldots ,N_{n}{]}^{T}$$

where, n is the number of satellites, x,y,z are the coordinate of station estimated as constants, dt is the clock error of receiver, Trop is the wet troposphere delay (ZWD), N is the integer carrier phase ambiguities estimated as float values. The clock errors of satellites are corrected by the IGS final clock product, the dry troposphere delay is corrected by Saastamoinen model, and the ionospheric delays are corrected respectively by the POLY model and the SVM-P model.

**Figure 9:**
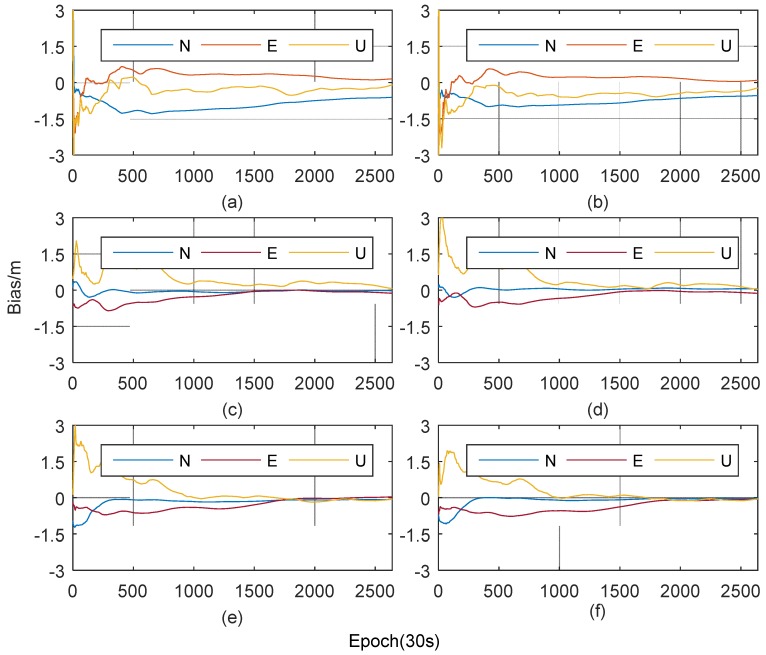
Single-frequency PPP results. (**a**,**c**,**e**) Station BTRD with no ionosphere model, the POLY and the SVM-P. (**b**,**d**,**f**) Station GTBH with no ionosphere model, the polynomial (POLY) and the Support Vector Machine (SVM-P.)

The observations C1 and L1 are selected, and Kalman filter is used as parameters estimator (engine) for static ambiguity-float PPP solution. The positioning results are shown in Figure 9.

The RMSs of positioning errors with different ionosphere models are listed in Table 3. These statistics are derived from the position residuals in PPP. It can be seen from Figure 9 and Table 3 that the accuracies of single-frequency PPP are about 0.7 m when ionospheric delay errors are not corrected, which indicates the ionosphere affects the positioning accuracy. Both ionosphere models can improve the single-frequency PPP accuracy. The positioning errors are all better than 0.3 m and improve over 60%. 

In each of four PPP experiments with ionosphere models in Figure 9b–f, the convergence rate in North direction is fastest, and it needs a period of time in East direction. And in Up direction, the trends of errors with two models are not consistent because of the effect of the ionospheric delay. When using the POLY model, due to the insufficient accuracy of ionospheric delay correction, the positioning errors in the Up direction fluctuated greatly, especially around the 1800 epoch. While, when using the SVM-P model, the errors in Up direction can remain stable after convergence. It can be seen from Table 3 that the RMSs of the two stations in the horizontal component are basically the same, but the difference of the RMSs in vertical component (Up direction) is quite obvious. This shows that two ionosphere model mainly affect the Up direction of the positioning. Since the RMSEs of the SVM-P model are lower than that of POLY and those are relatively stable in one day, the positioning accuracies of single-frequency PPP with the SVM-P are 0.141 m and 0.135 m, which increased by 47.6% and 40.2% than those using the POLY. Therefore, the PPP experiments prove that the SVM-P model can effectively improve the accuracy of ionospheric delay correction, thus improving the accuracy of single-frequency PPP positioning.

## 4. Discussion

**Figure 10:**
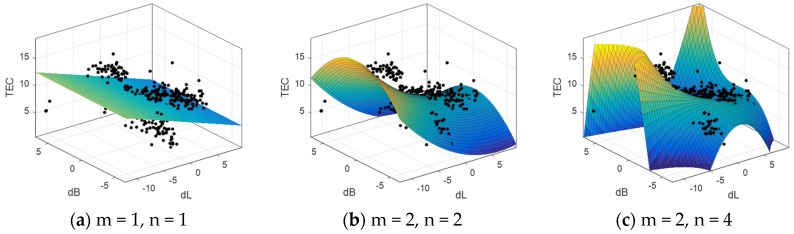
Ionospheric total electron content (TEC) modeling with different degree of polynomial.

According to the SVM-P method proposed in this paper, good performance is demonstrated in the above experiments. Because the distribution of the TEC is complex and irregular, when using the linear model for regional TEC fitting, the model will be affected by the overall trend of the TEC and ignore a lot of small-scale information. However, it is not appropriate to improve the order of linear model excessively to meet the high accuracy requirement for fitting, which will lead to more complicated calculations and the quite poor accuracy of the edge of the modeling area. Three sets of polynomial modeling were carried out according to the different parameters of the polynomial. The fitting results are as follows (Figure 10):

When using the 1st order polynomial, the estimated parameters are not enough to reflect the TEC information effectively; when using the 2nd order polynomial, the model can reflect the overall TEC distribution in the region; when a higher order is used, the more parameters increase the fitting accuracy of the regional center. However, it is severely distorted at the edge of the area, and even a large number of negative values appear. So, the polynomial parameters are chosen as n = m = 2.

SVM can achieve good nonlinear regression performance and the RBF kernel is used in the SVM algorithm. The POLY model residual is regarded as useful information instead of noise. The residual model is further fitted by SVM regression on the basis of the POLY model, and regional TEC model will be established combining the SVM and POLY. The RMSE comparisons of the TEC model at 23 hourly moments show that the model accuracy of the SVM-P is better than that of the POLY, which proves the feasibility of the above algorithm.

In addition, after the kernel parameters of the SVM are selected, the same results can be obtained by multiple experiments. However, due to the random assignment of weights, BPNN has different experimental results every time, which does not lead to satisfactory results in practical applications. In this paper, the optimal results of the BPNN-P are selected by multiple experiments.

However, there are some limitations to the SVM-P. In practical applications, we use the CORS data as a training set and the rover users as a test set. The training process of the SVM-P model requires a large number of observations, so it is suitable for the area with a sufficient number of GNSS stations. In spatial, the distribution of the CORS should be able to cover the total observation region and include the user’s range of motion. The number of training samples should not be less than the number of sample features. Besides, the variation of the ionosphere is correlated with the diurnal and seasonal changes, the selections of SVM kernel parameters are not unalterable, which should be chosen for different situations. Additionally, the SVM-P model is a post-processing method, and there is a delay when the ionospheric correction provided to real-time users, resulting in a loss of accuracy. Due to the small change of the ionosphere within a short time, the influence of time delay can be reduced by increasing the sampling rate of modeling. In the active period of the ionosphere, the interval of modeling can be decreased to 3-5min; and the interval can be increased to 15-30min in the quiet period.

## 5. Conclusions

In this paper, we combined the support vector machine regression algorithm and the 2D polynomial model for regional ionosphere modeling. The observations from JSCORS are used to train SVM, establish SVM-P model, and conduct static single-frequency PPP experiment. By using the grid search method, the optimal parameter combination of the SVM kernel parameters (C,γ) is selected. The analysis of the RMSE proves that the accuracy of the SVM-P model is 0.980 TECU, which is better than the polynomial model (1.185 TECU), with an improvement of 17.3%. Moreover, compared with the BPNN-P (1.070 TECU), the SVM-P model can display accurately the distribution of regional TEC. And in the single-frequency PPP experiment, the SVM-P model can effectively reduce the ionospheric delay error, and the positioning accuracies are increased by 47.6% and 40.2% compared with the polynomial model. 

For future works, we intend to optimize the selection of the SVM kernel parameters to reduce the search time and extend the modeling area to a wider range. At this time, the curvature of the earth’s surface should be taken into account and the spherical harmonic function may show great advantages compared to the polynomial [32].

## Figures and Tables

**Table 1:** Part of Support Vector Machine (SVM) training samples.

No.	Target	Input Parameters								
	$v_{POLY}$	$VTEC_{POLY}$	Δφ	ΔS	$\Delta \varphi^{2}$	$\Delta S^{2}$	ΔφΔS	$\Delta \varphi^{2}\Delta S$	$\Delta \varphi \Delta S^{2}$	$\Delta \varphi^{2}\Delta S^{2}$
1	−2.585	15.570	−0.075	−1.434	0.006	2.055	0.107	−0.008	−0.153	0.011
2	1.470	17.096	−1.473	1.628	2.171	2.651	−2.399	3.535	−3.906	5.755
3	−0.377	13.025	2.168	6.113	4.699	37.373	13.252	28.727	81.015	175.620
4	−1.372	15.359	−0.364	3.887	0.133	15.112	−1.415	0.515	−5.502	2.003
5	0.361	12.802	3.166	−2.952	10.025	8.712	−9.345	−29.589	27.583	87.332
6	−0.825	16.234	−1.208	−1.319	1.459	1.739	1.593	−1.924	−2.101	2.537
7	−0.225	13.928	1.640	−2.500	2.688	6.248	−4.098	−6.718	10.243	16.793
8	2.427	16.083	0.283	0.578	0.080	0.335	0.164	0.046	0.095	0.027
9	0.313	12.459	3.919	5.080	15.360	25.804	19.909	78.027	101.131	396.357
10	−0.262	15.005	1.413	2.878	1.998	8.282	4.067	5.749	11.705	16.543
11	0.162	11.574	4.701	−3.926	22.095	15.413	−18.454	−86.742	72.450	340.549
12	−0.691	17.145	−3.044	−1.110	9.264	1.233	3.379	−10.285	−3.752	11.419
13	0.407	14.961	−0.223	−2.317	0.050	5.370	0.516	−0.115	−1.196	0.267
14	1.585	17.264	−1.592	0.720	2.535	0.518	−1.146	1.825	−0.825	1.314
15	0.264	13.575	2.122	5.224	4.502	27.294	11.085	23.521	57.913	122.881
16	0.292	16.030	−0.464	2.978	0.215	8.867	−1.381	0.641	−4.113	1.908
17	−0.177	12.502	2.972	−3.837	8.833	14.725	−11.405	−33.895	43.764	130.068
18	−1.153	16.146	−1.886	−1.744	3.557	3.041	3.289	−6.202	−5.735	10.816
19	−1.420	13.952	0.934	−2.940	0.873	8.642	−2.746	−2.566	8.074	7.543
20	0.787	16.471	−0.410	0.111	0.168	0.012	−0.045	0.019	−0.005	0.002

**Table 2:** Root mean square errors (RMSEs) of three ionosphere models in different moments (TECU).

UTC	POLY	BPNN-P		SVM-P	
	RMSE	RMSE	Improvement	RMSE	Improvement
1:00	1.154	1.123	2.7%	1.019	11.7%
2:00	1.424	1.156	18.8%	1.161	18.5%
3:00	1.670	1.009	39.6%	0.964	42.3%
4:00	1.605	1.229	23.5%	1.301	19.0%
5:00	1.206	1.235	−2.4%	1.112	7.8%
6:00	1.552	1.297	16.4%	1.240	20.1%
7:00	1.117	0.987	11.7%	0.931	16.7%
8:00	0.897	0.801	10.7%	0.738	17.7%
9:00	0.977	0.918	6.0%	0.838	14.2%
10:00	1.032	0.917	11.1%	0.912	11.6%
11:00	0.907	0.928	−2.3%	0.817	9.9%
12:00	0.902	0.835	7.3%	0.803	10.9%
13:00	0.937	0.970	−3.5%	0.832	11.2%
14:00	1.031	1.044	−1.2%	0.918	11.0%
15:00	1.272	1.164	8.5%	1.076	15.4%
16:00	1.189	1.282	−7.8%	1.103	7.2%
17:00	0.972	0.971	0.1%	0.879	9.6%
18:00	1.377	1.326	3.7%	1.050	23.8%
19:00	1.575	1.138	27.8%	1.095	30.5%
20:00	1.420	1.311	7.7%	1.072	24.5%
21:00	0.886	0.927	−4.6%	0.828	6.6%
22:00	1.233	1.055	14.4%	0.996	19.2%
23:00	0.908	0.995	−9.6%	0.853	6.1%
Mean	1.185	1.070	9.6%	0.980	17.3%

**Table 3:** Results of single-frequency PPP (RMS/m).

Station	Model	North	East	Up	3D	Improvement
BTRD	None	0.680	0.176	0.256	0.747	-
	POLY	0.020	0.067	0.259	0.269	64.0%
	SVM-P	0.082	0.019	0.113	0.141	81.2%
GTBH	None	0.599	0.100	0.385	0.719	-
	POLY	0.060	0.074	0.204	0.225	68.7%
	SVM-P	0.032	0.091	0.094	0.135	81.3%
